# Validation of a computer-adaptive test to evaluate generic health-related quality of life

**DOI:** 10.1186/1477-7525-8-147

**Published:** 2010-12-03

**Authors:** Pablo Rebollo, Ignacio Castejón, Jesús Cuervo, Guillermo Villa, Eduardo García-Cueto, Helena Díaz-Cuervo, Pilar C Zardaín, José Muñiz, Jordi Alonso

**Affiliations:** 1 BAP Health Outcomes Research, Calle Azcárraga 12 A, 33010, Oviedo, Spain; 2 Universidad de Oviedo, Psychology Department, Plaza Feijoo s/n, 33003, Oviedo, Spain; 3 Institut Municipal d'Investigació Mèdica (IMIM-Hospital del Mar), Doctor Aiguader 88, 08003, Barcelona, Spain; 4 CIBER en Epidemiología y Salud Pública (CIBERESP), Doctor Aiguader 88, 08003, Spain

## Abstract

**Background:**

Health Related Quality of Life (HRQoL) is a relevant variable in the evaluation of health outcomes. Questionnaires based on Classical Test Theory typically require a large number of items to evaluate HRQoL. Computer Adaptive Testing (CAT) can be used to reduce tests length while maintaining and, in some cases, improving accuracy. This study aimed at validating a CAT based on Item Response Theory (IRT) for evaluation of generic HRQoL: the CAT-Health instrument.

**Methods:**

Cross-sectional study of subjects aged over 18 attending Primary Care Centres for any reason. CAT-Health was administered along with the SF-12 Health Survey. Age, gender and a checklist of chronic conditions were also collected. CAT-Health was evaluated considering: 1) feasibility: completion time and test length; 2) content range coverage, Item Exposure Rate (IER) and test precision; and 3) construct validity: differences in the CAT-Health scores according to clinical variables and correlations between both questionnaires.

**Results:**

396 subjects answered CAT-Health and SF-12, 67.2% females, mean age (SD) 48.6 (17.7) years. 36.9% did not report any chronic condition. Median completion time for CAT-Health was 81 seconds (IQ range = 59-118) and it increased with age (p < 0.001). The median number of items administered was 8 (IQ range = 6-10). Neither ceiling nor floor effects were found for the score. None of the items in the pool had an IER of 100% and it was over 5% for 27.1% of the items. Test Information Function (TIF) peaked between levels -1 and 0 of HRQoL. Statistically significant differences were observed in the CAT-Health scores according to the number and type of conditions.

**Conclusions:**

Although domain-specific CATs exist for various areas of HRQoL, CAT-Health is one of the first IRT-based CATs designed to evaluate generic HRQoL and it has proven feasible, valid and efficient, when administered to a broad sample of individuals attending primary care settings.

## Background

Health-Related Quality of Life (HRQoL) is among the most used variables in Health Outcomes Research (HOR) in the academic field, as well as in clinical trials and post-authorisation studies. It refers to the subjective valuation of the influence of health on the individuals' ability of having a normal functioning which makes it possible to perform all the activities which are important for them and which affect their well-being [1]. Although during the last 35 years HRQoL assessment has had an enormous development worldwide, several barriers limit its use in the clinical practice. These barriers had been described by Deyo and Patrick in 1989 [2] and were revised in Spain in 2005 [3]. Classical Test Theory (CTT) cannot solve certain practical issues, such as the high number of questions needed to complete a multi-dimensional HRQoL questionnaire and the lack of accuracy when dealing with the change of individual scores over time. Item Response Theory (IRT) [4,5] overcomes some of the limitations that may affect instruments developed under the CTT. CAT instruments based on IRT, clearly increase "measurement efficiency" (the ratio of a measure's psychometric soundness to the response burden the measures imposes). A greater measurement precision can be achieved through the selection of a few items from a calibrated item pool that combines high quality items from multiple instruments into a single data resource [6]. Since each item is independently described by parameters such as difficulty or discrimination [7], they can be combined as necessary, therefore, there is no questionnaire as such but different combinations of items which provide comparable scores. Through IRT, an Item Characteristic Curve (ICC) is constructed for each item; this curve reflects the probability of the answer to each item for each HRQoL level. Using ICC, the HRQoL level of a given subject can be estimated after answering any number of items. Furthermore, IRT allows us to estimate the contribution each item makes to the assessment for each level of the variable: the Information Function. Measurement error is inversely linked to the information used and hence an error estimate is available for each assessment.

Based on this theory, Computer Adaptive Tests (CATs) arise as a psychometric assessment technique administered through a computer. For each respondent, the selection of items is adapted to the prior estimates of the construct being assessed [7]. These tests have been successfully used in Education and Psychology fields [8] and they allow a more practical assessment and a more accurate estimation of the variable being measured, in this case, HRQoL. CATs result in the individual administration of questionnaires, as well as in the collection and computation of responses, providing instant results [9].

Since the 90 s, when some authors recommended CAT applications for health [10], a variety of CATs have been developed in the Health field, as those for migraine [11,12], rheumatoid arthritis [13], osteoarthritis [14], back pain [15], physical therapy [16,17], anxiety [18], cancer [19] and paediatrics [20]. All these CATs focus on one specific condition or HRQoL domain, but they cannot measure generic HRQoL in healthy or ill subjects from the general population. As it is well known, HRQoL is essentially a multidimensional concept. This fact could make it difficult to accomplish the unidimensionality required for the application of IRT [21]. Despite this fact, we think it is possible to develop a calibrated item pool to measure the underlying construct of generic HRQoL. Pursuing this aim, in a previous study [22], an expert panel proposed a pool of 140 five-level Likert items, chosen among several HRQoL questionnaires validated in Spain. That pool was first administered to a pilot sample and later to a general sample of patients belonging to 7 Primary Health centres. Two administration options were offered to the later sample: 1) on paper 2) on a touch panel. Item Response Theory psychometric properties (discrimination, reliability and validity) were evaluated by means of a Factorial Analysis and other methods. The Information Function was analyzed and an application method was tested by means of simulation: a minimum of 5 items and a maximum of 15 were shown; the first item was randomly selected among 13 which deal with generic health aspects and covered a broad HRQoL range. These 13 initial items were selected among the most informative items by an expert panel. The result was a calibrated pool of 96 items [22]. This pool of items showed a factorial structure in one dimension (with 45% of the variance explained and a lowest loading of 0,224) and evidenced high reliability (Cronbach's alfa = 0.99).

This manuscript presents the validation of CAT-Health: a CAT based on the described calibrated item pool, using the mentioned application method and implemented with a touch screen interface. This validation study, gathering information from a sample of subjects from the Spanish general population, pursues the goal of obtaining a feasible and accurate instrument to measure generic HRQoL in the clinical setting.

## Methods

A cross-sectional study of subjects aged over 18 attending one of the four participant Primary Care Centres (PCC) for any reason was carried out, in order to assess the validity of CAT-Health. The study hence included subjects with chronic conditions or acute pathologies but also healthy subjects, for instance, patients' healthy relatives at the PCC. Before their inclusion, all patients were informed and provided written informed consent, in accordance with the ethical principles of the Declaration of Helsinki and the Good Clinical Practice guidelines.

Data were collected during three consecutive days in each PCC, between February and March 2007, by using two methods: a tablet PC (electronic pencil required) and a touch screen panel (neither mouse nor pencil necessary). Subjects completed a very short initial questionnaire about their age, sex and whether they had suffered any of the chronic conditions presented in a checklist including: anxiety, depression, acute disease, arterial hypertension, cardiac disease, diabetes, joint pain, migraine, pulmonary disease and "other diseases". The patients filled this chronic conditions checklist on their own. In addition, the SF-12 Health Survey was administered using the same devices [23-25]. CAT-Health and SF-12 completion times were automatically recorded.

### The CAT-Health system

The CAT-Health system evaluates the generic HRQoL of healthy or ill subjects from the general population by showing a variable number of items (between 5 and 15), extracted from a unidimensional calibrated pool of 96 items which had been previously developed [22]. All the items in the pool have 5 response categories. The first item is randomly selected among 13 initial items which cover a broad range of the measured construct (HRQoL) and which focus on generic health aspects. These 13 initial items were selected among the most informative items by an expert panel Based on the response to this first question, the system selects the most informative item to be presented as the following question, iteratively. The system stops when either: 1) the maximum number of items (n = 15) has been presented to the subject; or 2) the minimum number of items has been achieved, the estimation error is below the unity and the percentage of reduction of the error, with respect to the previous estimation, is below 5%. This application method was theoretically tested by means of a simulation study [22] and it provided an accurate score for moderately low or high HRQoL levels (near to general population mean score). The score calculated by CAT-Health system has a theoretical range between -3.85 and +3.87, but it was normalized to a 50 ± 10 distribution, in order to facilitate its interpretation: (CAT score - mean)/SD * 10 + 50. The higher the score, the better the HRQoL.

### Evaluation of the CAT-Health system

CAT-Health was evaluated considering three different criteria: 1) feasibility of the system in the clinical practice, in terms of completion time and test length, paying special attention to elderly subjects (fourth quartile of the distribution). 2) Psychometric evaluation, including content range coverage, Item Exposure Rate (IER) and test precision; and 3) Validity assessment of CAT-Health. Construct validity was studied through the analysis of differences in CAT-Health scores depending on sex (females were expected to have worse scores than males), age (elderly people were expected to show worse scores) and the presence of reported chronic conditions (the higher the number of conditions, the worse the score). It was also hypothesized that subjects with one of the listed pathologies should have a worse score than subjects without them. Migraine, acute pathology, hypertension and "other pathologies", however, were not considered to be associated to HRQoL a priori, because the content of the items was not designed to take into account acute or silent pathologies. Finally, CAT-Health convergent validity was evaluated with respect to SF-12 physical and mental component scores. A moderate correlation between both questionnaires was expected (correlation coefficients between 0.3 and 0.6), as it is usually found when HRQoL generic questionnaires are compared [26-28]

### Statistical analysis

Absolute and relative frequencies were used to describe the sample distribution with respect to the nominal variables (sex and declared pathologies). Mean and standard deviation were used for the continuous variables (age, CAT-Health and SF-12 scores, and number of declared pathologies). Quartiles were used in the case of the CAT-Health completion times and test length. Differences in the number of items shown to subjects, according to age and chronic conditions, were assessed by means of a Kruskal-Wallis test. Differences according to sex were evaluated by means of a Mann-Whitney test.

Content range coverage of CAT-Health was studied through the analysis of floor and ceiling effects. Item Exposure Rate (IER) was defined as the ratio of the total number of times a given item is shown to the number of times CAT-Health was administered. Test precision was studied through the analysis of the Test Information Function (TIF), which is an aggregate of the information provided by each item. Considering the adaptive nature of the system and that the number of items shown within each test were limited to 15, a TIF using all the items in the pool is not representative of a typical test, so a TIF was constructed by using just the 15 items which provided more information at each level (given this was a continuous range, a process of discretization was necessary and 800 HRQoL levels were considered). Note that the selection of items might change within the HRQoL range.

Differences in CAT-Health scores between males and females and between subjects who declared having any of the listed pathologies were assessed by means of a *t *test. Lineal regression analysis was performed to confirm the association of each of the pathologies independently with the CAT-Health score. Correlation between CAT-Health scores and age and the number of pathologies was analyzed by means of Pearson and Spearman correlation coefficients, respectively. The sample was divided into 4 groups, according to the number of declared pathologies (none, 1, 2, 3 or more) and also according to the quartiles of the distribution of age; CAT-Health and SF-12 scores were compared among these groups by means of One-Way ANOVA. Effect size was computed for CAT-Health score and the physical and mental components of SF-12, when comparing mean differences between groups (age, sex and number of pathologies): Cohen's *d *in the case of a *t *test and *eta square *in the case of ANOVA. Following the guidelines proposed by Cohen [29], for *t *tests, an effect size of 0.1 was considered small, 0.3 was considered medium and 0.5 was large. In the case of ANOVA, 0.01, 0.06 and 0.14 were considered small, medium or large, respectively.

Correlations between the CAT-Health score and the SF-12 physical and mental components were assessed by means of Pearson correlation coefficients.

## Results

The characteristics of the sample (N = 396) are described in Table 1. The mean age (SD) was 48.6 (17.7) years and two thirds of the subjects were female. Moreover, one third of the participants did not report any chronic condition from the list.

**Table 1:** Sample description (N = 396)

Sample description			
	All (N = 396)	Male (N = 130)	Female (N = 266)
**Mean age (SD)**	48.61 (17.67)	49.03 (18.45)	48.40 (17.30)
**Frequency of declared pathologies: n (%)**			
**Anxiety**	70 (17.7%)	11 (8.5%)	59 (22.2%)
**Depression**	56 (14.1%)	5 (3.8%)	51 (19.2%)
**Acute pathology**	98 (24.7%)	31 (23.8%)	67 (25.2%)
**Arterial hypertension**	66 (16.7%)	23 (17.7%)	43 (16.2%)
**Cardiac disease**	32 (8.1%)	13 (10%)	19 (7.1%)
**Diabetes**	37 (9.3%)	19 (14.6%)	18 (6.8%)
**Joint pain**	125 (31.6%)	32 (24.6%)	93 (35%)
**Migraine**	36 (9.1%)	5 (3.8%)	31 (11.7%)
**Pulmonary disease**	31 (7.8%)	13 (10%)	18 (6.8%)
**Other pathology**	85 (21.5%)	34 (26.2%)	51 (19.2%)
**Number of self-reported conditions**			
**0**	36.9%	43.1%	33.8%
**1**	33.8%	36.2%	32.7%
**2**	16.4%	12.3%	18.4%
**3 or more**	12.9%	8.4%	15.1%

Regarding the analysis of the practical use of CAT-Health, the median completion time of CAT-Health was 81 seconds (IQ range = 59 seconds-118 seconds), increasing with age (p < 0.001) from 66 seconds in the group of subjects aged under Pc25 to 107 seconds in the group of subjects aged over Pc75. The median number of the CAT-Health items shown to subjects was 8 (IQ range = 6-10). There were not statistically significant differences (p > 0.05) in the number of items shown to males and females, to subjects from different age groups and to subjects according to the number of declared pathologies.

The CAT-Health non-normalized score ranged between -2.4 and +2.8 (theoretical range between -3.8 and +3.8). Neither ceiling nor floor effects were found. The mean normalized score was 50.88 (6.02), with a minimum of 34.71 and a maximum of 80.89. The mean Physical Component Summary (PCS) score of SF-12 was 46.84 (10.1) and it ranged between 19.12 and 67.06; the mean Mental Component Summary (MCS) score was 46.88 (10.96), ranging between 11.18 and 67.83.

**Figure 1:**
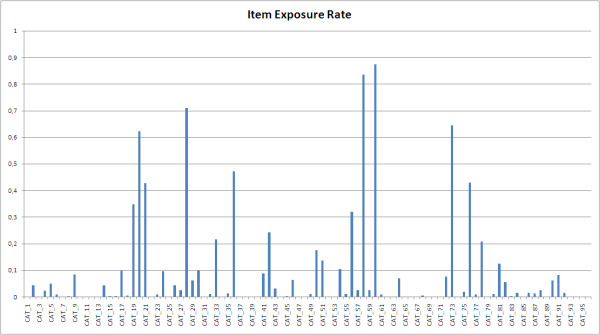
**The CAT-Health Item Exposure Rate (IER)**. The IER is the ratio of the total number of times one item is presented to the number of times CAT-Health is administered.

**Figure 2:**
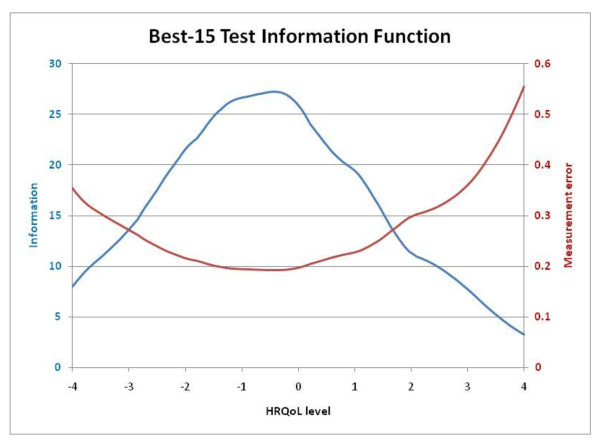
**The CAT-Health best-15 Test Information Function (TIF)**. The TIF is used to evaluate the test precision for different HRQoL levels.

Figure 1 shows CAT-Health Item Exposure Rate (IER). None of the items in the pool had an exposure rate of 100%, while 36 items (37.5%) were not shown at any time (20 of them could not be shown with the chosen application method). IER was over 5% for 26 items (27.1%). The best-15 TIF is shown in Figure 2 along with the standard error for each level of the scale. The Test Information Function (TIF) peaked between level -1 and 0 of HRQoL, which corresponds to the normalized scores 35.58 and 52.19. In this part of the scale, the error was below 0.2.

Regarding construct validity, in Table 2 the comparison of CAT-Health and SF-12 scores is shown with respect to sex, age and the number of declared pathologies. CAT-Health and SF-12 MCS scores were higher for males than for females (p < 0.0001). The effect size (ES) of CAT-Health (0.46) was similar to that of MCS (0.51) and higher than that of PCS (0.12). The CAT-Health score showed a negative statistically significant correlation with age (r = -0.351; p < 0.001) as SF-12 PCS did (r = -0.255; p < 0.001); SF-12 MCS did not show a statistically significant correlation with age. By dividing the sample in four age groups (under 34.27 years; 34.27-46.26; 46.27-61.19; over 61.19), ANOVA analysis showed statistically significant differences (p < 0.0001) among age groups for CAT-Health and PCS scores. The CAT-Health score also showed a negative statistically significant correlation with the number of pathologies declared by the respondents (r = -0.548; p < 0.01), like PCS and MCS (r = -0.337 and r = -0.262, respectively; p < 0.01). The ES of CAT-Health (0.12) was higher than those of PCS (0.06) and MCS (0.01).

**Table 2:** Comparison of the CAT-Health and SF-12 scores according to sex, age and number of self-reported conditions

Comparison of the CAT-Health and SF-12 scores according to sex, age and number of declared pathologies				
		**CAT-Health**	**SF-12 PCS**	**SF-12 MCS**

**Sex**	Male (N = 130)	52.68 (6.51)	47.65 (9.43)	50.89 (9.45)
	Female (N = 266)	49.99 (5.56)	46.44 (10.40)	44.92 (12.57)
	p (*t *test)	< 0.0001	0.262	< 0.0001
	Effect size (Cohen's *d*)	0.455	0.120	0.513

**Age**	< 34.27 years (N = 100)	53.96 (5.69)	50.46 (7.98)	47.59 (11.19)
	34.27-46.26 years (N = 98)	51.33 (5.53)	47.82 (10.42)	47.24 (11.65)
	46.27-61.19 years (N = 99)	49.66 (5.29)	44.83 (10.32)	45.36 (12.30)
	> 61.19 years (N = 99)	48.54 (6.18)	44.20 (10.36)	47.34 (12.70)
	p (ANOVA)	< 0.0001	< 0.0001	0.537
	Effect size (*eta *square)	0.116	0.062	0.006

**Number of self-reported conditions**	None (N = 146)	54.23 (5.72)	50.56 (7.84)	50.80 (9.04)
	1 pathology (N = 134)	50.89 (4.86)	46.74 (10.23)	46.52 (12.40)
	2 pathologies (N = 65)	47.75 (7.42)	44.14 (10.81)	42.78 (12.70)
	3 or more pathologies (N = 51)	45.24 (4.66)	39.85 (9.97)	41.86 (13.59)
	p (ANOVA)	< 0.0001	< 0.0001	< 0.0001
	Effect Size (*eta *square)	0.272	0.124	0.082

**Figure 3:**
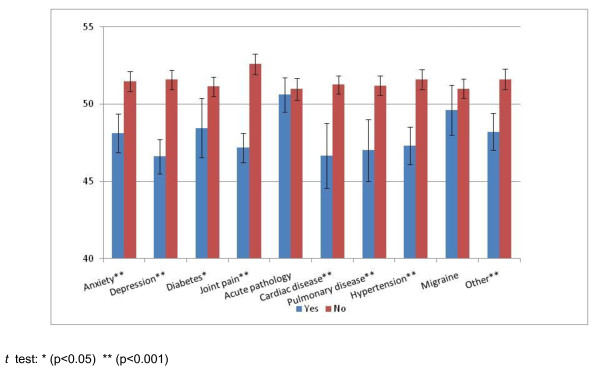
**Differences in the CAT-Health scores according to self-reported conditions (means and 95% confidence intervals)**.

Figure 3 shows the CAT-Health scores of subjects who declared suffering from any of the listed pathologies and of those who did not. Differences were all statistically significant (p < 0.05), except that of acute pathology and migraine. These differences remained statistically significant when analysing each of the pathologies adjusting by the rest of them in a regression analysis. SF-12 PCS did not detect differences between patients with or without anxiety, depression, acute disease, pulmonary disease and migraine; neither did SF-12 MCS detect differences in the case of diabetes, acute disease, cardiac disease, pulmonary disease, arterial hypertension and "other disease".

The correlation coefficients between CAT-Health scores and SF-12 PCS (r = 0.547) and MCS (r = 0.346) were moderate and statistically significant (p < 0.001).

## Discussion

Several computer-adaptive tests (CAT) evaluating health outcomes have been developed and validated in recent years, but, to our knowledge, the CAT-Health system is one of the first CATs designed to evaluate generic HRQoL. The results of the present study show that CAT-Health is feasible, valid and efficient for HRQoL evaluation: its psychometric properties were satisfactory when evaluating HRQoL in a wide range of subjects attending primary care settings.

The use of HRQoL as a health outcome measure is becoming more important in the evaluation of patient care and health services. It is usually evaluated using questionnaires based on CTT, administered through pencil and paper. Once the answers of a subject are collected, the questionnaire has to be coded and scored, and then the results have to be interpreted. This is a time consuming process and, therefore, it is expensive, especially for follow-ups in clinical practice. These problems constitute a barrier that prevents the evaluation of HRQoL in the clinical setting [3]. CAT instruments clearly increase "measurement efficiency" (the ratio of a measure's psychometric soundness to the response burden the measures imposes) [7]. CAT instruments decrease the response burden, diminishing the number of questions to be answered by the subject; they have small floor and ceiling effects, when they use an extensive item bank; they reduce the error of measurement and so they measure much more accurately; they are flexible, adapting themselves to the trait level of the respondent and also to specific measurement contexts [11-20].

Historically, HRQoL has been considered a multi-dimensional concept. Some authors argue, however, that there is an underlying construct that affects all them and can be directly measured [30]. In a previous paper, we presented the development and calibration of a generic HRQoL item bank, so that the assessment of HRQoL with a single dimension was feasible [22]. Using that item bank, we developed one of the first IRT-based CATs for the evaluation of generic HRQoL, the validation of which we have presented in this manuscript.

The evaluation of CAT-Health has shown that CAT-Health system median completion time is really short (under 1 minute and a half). Even though we have shown that the completion time was associated with the age of the respondents (it must be taken into account that elderly people are not familiar with the interfaces used to fill in the questionnaire), the median completion time for the group of individuals in the last quartile of age (over 61.19 years) was still well beneath 2 minutes. Also, the evaluation of generic HRQoL with CAT-health required a median of 8 items, a test length similar to that of published CATs for specific groups of pathologies: AM-PAC-CAT [16], for post-acute care, showed a mean of 6.6 items; each of the 5 domains of the CAT-5D-QOL [15], for back pain, 4.4 to 6.6 items; and the Anxiety-CAT [18] 6 to 8 items.

In this validation study, 60 items were shown at least once, which represents the 79% of the items in the pool that could be shown, according to the chosen application method (at least 20 items will never appear, as they are not amongst the 15 most informative for any HRQoL level). The fact that none of the items had an IER of 100% and that the number of different items used at some point was high indicates that the system actually adapted the items presented to the individuals in the sample, pointing to the adequacy of a CAT for HRQoL measurement.

With respect to the content range coverage of CAT-Health, the frequency distributions of the non-normalized scores of the subjects under study were normally distributed, with no floor nor ceiling effects, as expected for a generic instrument in this sample. On the contrary, the abovementioned domain-specific CATs presented ceiling effects: 10% for AM-PAC-CAT [16] (that presented a roughly normally distributed score) and 0% to 6.1% for the different CAT-5D-QOL scales [15]. Normalized CAT-Health scores (50 ± 10) had a similar range to those of SF-12 PCS and slightly narrower than those of MCS. The performance of CAT-Health in HRQoL assessment in the studied sample, which covered a broad spectrum of individuals attending primary care settings, indicates a good potential for the evaluation of the general population.

The analysis of the TIF showed that CAT-Health is a very discriminative measurement tool in the range of scores between 35 and 52, which corresponds to a normal or slightly deteriorated HRQoL, the most frequent status in people who demand health care at a primary care centre. Future research is needed in order to add new items to the pool which would allow CAT-Health to cover a broader range of HRQoL.

The analysis of validity demonstrated that the CAT-Health score is a valid generic measure of HRQoL. CAT-Health adequately detects the hypothesized differences between male and female subjects, as well as between different age groups and among groups by the number of declared pathologies.

Recently, the Adaptive Measurement of Change (AMC) has been proposed as a feasible and effective method for measuring individual change using CATs [31]. The availability of an error estimate for each subject, in this type of measurements, turns high the precision of CATs, like CAT-Health, into useful instruments for monitoring HRQoL. The sensitivity to change of CAT-Health will be addressed in the future by means of a longitudinal study. The validation of the system in a broader random sample of the general population and the adaptation for its use in English are also planned. These studies will include a larger number of clinical variables to allow for a detailed evaluation of the construct validity of the system.

## Conclusions

Although domain specific CATs exist for various areas of HRQoL, CAT-Health is one of the first IRT-based CAT designed to evaluate generic HRQoL and it has proven feasible, valid and efficient, when administered to a broad sample of individuals attending primary care settings.

The reduced number of items required for HRQoL evaluation and the resulting shortened completion time, together with the characteristics inherent to computerized instruments, such as automatic scoring and interpretation of results, make of CAT-Health a practical instrument for clinical settings, as Primary Care Centres. These two facts, along with its sound psychometric properties, which open the possibility of evaluating HRQoL changes at the individual level, are important advantages of the CAT-Health system over other generic questionnaires based on CTT.

## Competing interests

The authors hereby declare that there is no conflict of interests, financial agreement or other involvement with any company whose product figures in the submitted work. Pablo Rebollo, Ignacio Castejón, Jesús Cuervo, Guillermo Villa and Helena Díaz-Cuervo work at BAP Health Outcomes Research, which is the Applicant of a Spanish patent application (P200701072) and a European patent application (EPO8013312) related to the CAT-Health system.

## Authors' contributions

All authors are responsible for the reported manuscript and have participated in its concept and design, analysis and interpretation of data and, finally, in its drafting and review.

## Note

The Spanish C.A.T-Health group is formed by researchers from:

BAP Health Outcomes Research, Oviedo, Spain; 4^th ^Area Primary Care Centers, Principality of Asturias Health Service, Oviedo, Spain; Universidad de Oviedo, Psychology Department, Oviedo, Spain; Universidad Complutense de Madrid, Methodology Department, Madrid, Spain; Institut Municipal d'Investigació Mèdica (IMIM-Hospital del Mar), Health Research Unit Services, Barcelona, Spain.
